# Solid-Phase Synthesis Approaches and U-Rich RNA-Binding Activity of Homotrimer Nucleopeptide Containing Adenine Linked to *L*-azidohomoalanine Side Chain via 1,4-Linked-1,2,3-Triazole

**DOI:** 10.3390/ijms262311687

**Published:** 2025-12-02

**Authors:** Piotr Mucha, Małgorzata Pieszko, Irena Bylińska, Wiesław Wiczk, Jarosław Ruczyński, Piotr Rekowski

**Affiliations:** 1 Laboratory of Biologically Active Compounds Chemistry, Department of Molecular Biochemistry, Faculty of Chemistry, University of Gdansk, Wita Stwosza 63, 80-308 Gdansk, Poland; gosia.pieszko@gmail.com (M.P.); jaroslaw.ruczynski@ug.edu.pl (J.R.); piotr.rekowski@ug.edu.pl (P.R.); 2 Environmental Nucleic Acid Laboratory, Faculty of Chemistry, University of Gdansk, Wita Stwosza 63, 80-308 Gdansk, Poland; 3 Laboratory of Photobiophysics, Department of Biomedical Chemistry, Faculty of Chemistry, University of Gdansk, Wita Stwosza 63, 80-308 Gdansk, Poland; irena.bylinska@ug.edu.pl (I.B.); wieslaw.wiczk@ug.edu.pl (W.W.)

**Keywords:** nucleobase amino acid, nucleopeptide, click chemistry, triazole, TAR RNA HIV-1, RNA–nucleopeptide interaction, tRNALys3 anticodon, solid phase peptide synthesis

## Abstract

Nucleopeptides (NPs) are unnatural hybrid polymers designed by coupling nucleobases to the side chains of amino acid residues within peptides. In this study, we present the synthesis of an Fmoc-protected nucleobase amino acid (NBA) monomer (Fmoc-1,4-TzlNBA_A_) with adenine attached to the side chain of *L*-homoazidoalanine (Aha) through a 1,4-linked-1,2,3-triazole. The coupling was accomplished by a Cu(I)-catalyzed azide–alkyne cycloaddition (CuAAC) of Fmoc-Aha and *N9*-propargyladenine. Subsequently, a homotrinucleopeptide (HalTzl_AAA_) containing three 1,4-TzlNBA_A_ residues was synthesized, using different solid-phase peptide synthesis (SPPS) approaches, and its ability to recognize U-rich motifs of RNAs involved in the HIV replication cycle was studied using circular dichroism (CD) and fluorescence spectroscopy. CD curves confirmed the binding of HalTzl_AAA_ to U-rich motifs of the transactivation responsive element (TAR UUU RNA HIV-1) bulge and the anticodon stem–loop domain of human tRNALys3 (ASLLys3) by a decrease in the positive ellipticity band intensity around 265 nm during the complexation. 5′-(FAM(6))-labeled TAR UUU and hASLLys3 were used for fluorescence anisotropy binding studies. Fluorescence data revealed that HalTzl_AAA_ bound TAR’s UUU bulge with a moderate affinity (K_d_ ≈ 38 µM), whereas the ASLLys3 UUUU-containing loop sequence was recognized with 2.5 times lower affinity (with K_d_ ≈ 75 µM). Both the standard SPPS method and its variants, which involved the attachment of adenine to the L-Aha side chain using the click reaction during the synthesis on the resin or after the nucleopeptide cleavage, were characterized by a similar efficiency and yield. The CD and fluorescence results demonstrated that HalTzl_AAA_ recognized the U-rich sequences of the RNAs with moderate and varied affinities. It is likely that both the hydrogen bonds associated with the complementarity of the interacting sequences and the conformational aspects associated with the high conformational dynamics of U-rich motifs are important in the recognition process. The nucleopeptide represents a new class of RNA binders and may be a promising scaffold for the development of new antiviral drugs.

## 1. Introduction

From the research conducted so far on the function of RNA in cells, a picture emerges in which RNA is a “main player” rather than a passive intermediary in processes related to the flow of genetic information [1,2]. Being a key factor responsible for the proper flow of genetic information, and thus maintaining cell homeostasis, RNA has emerged as a potential therapeutic target of modern pharmacology [3,4,5]. Due to the limitations of RNA backbone stability, constrained transport across cell membranes, and moderate affinity for target DNA/RNA sequences, numerous efforts have been made to obtain RNA-binding ligands with improved affinity, specificity, and other biochemical or pharmacological parameters [6,7]. Much effort has been put into the search for small-molecule modulators and inhibitors that, by recognizing unique RNA structures, would be able to control its activity [8,9].

NPs are artificial polymers designed by attaching nucleobases to the side chains of amino acid residues of peptide or peptide-like backbones [10,11]. This allows the structural features of nucleic acid and protein to be combined in a single molecule. NPs recognize RNA with high affinity and specificity [12,13,14]. Among the well-known examples are peptide nucleic acids (PNAs) [15,16,17], which contain nucleobases connected through an acetyl linker to a non-classical peptide chain consisting of *N*-(2-aminoethyl)glycine residues. PNA, due to its high specificity and nucleic acid recognition affinity, is currently the most efficient type of nucleopeptide designed to-date. However, it is burdened by poor solubility, a tendency to self-aggregate, and an inability to enter the cell by penetrating the cell membrane [18,19]. The plasticity and ease of modification of the classical peptide backbone, which is the core of the NPs’ structure, offer new possibilities of eliminating the disadvantages of PNA [20,21]. The ability of NPs to specifically recognize nucleic acids led to the idea of using them as inhibitors of HIV replication [11,12]. NPs have been demonstrated to be inhibitors of both viral RNAs and HIV proteins [22].

U-rich sequences play important roles in RNA activity, especially in its post-transcriptional regulation [23]. Many U-rich RNA motifs serve as critical cis-regulatory elements that recruit specific RNA-binding proteins to modulate mRNA stability and degradation, thereby influencing gene expression dynamics [23]. Therefore, U-rich RNA sequences like those located in the 3′ untranslated regions (UTRs) of mRNAs can be targeted for therapeutic purposes [24,25,26,27]. U-rich RNA sequences play a crucial role in HIV-1 infection, controlling various steps of the viral replication cycle [28]. As a result, several viral RNA fragments have garnered interest for their potential use as therapeutic targets [28,29]. One extensively studied RNA-based model is the *trans*-activation responsive region (TAR) RNA located at the 5′-end of nascent transcripts of HIV-1, responsible for elongating viral mRNAs during the early stage of transcription. TAR is one of the key regulatory structures of HIV [30,31]. When it is deleted or mutated, the transcription of proviral DNA integrated with a host is strongly inhibited or completely blocked. This ultimately prevents the formation of new viral particles and inhibits the infection [31]. TAR RNA features a 59 bp stem–bulge–loop structure [30,32,33]. The three-nucleotide bulge (UCU or UUU) interacts with an arginine-rich region (ARR) of the 86 to 102-amino acid Tat HIV-1 protein [34]. Short Tat peptides containing the ARR motif recognize the TAR RNA bulge with a specificity similar to that of the native HIV-1 Tat protein, albeit with a lower affinity [18,35,36,37].

During the assembly of the HIV-1 virion, human cytoplasmatic tRNAs specific for lysine (htRNALys) are selectively packaged into the virion, and only one of these, isoacceptor type 3, htRNALys3, is annealed to the viral ssRNA genome, where it primes reverse transcription of the viral genomic ssRNA into double-stranded proviral DNA, which is then integrated with the host chromosome [38]. The annealing of tRNALys3 to 5′-terminal regulatory sequences of viral RNA is a complicated multi-staged process [39]. Multiple fragments of the tRNA are involved in interaction with the viral RNA genome. One of them is the U-rich anticodon stem–loop domain of htRNALys3 (ASLLys3), which interacts with an A-rich internal loop located upstream to the priming binding side (PBS) loop [39]. The ASLLys3 U-rich loop is highly post-transcriptionally modified [40]. However, an unmodified tRNALys3 transcript is also active in the process of reverse transcription [41]. ASLLys3 U-rich loop interaction with the A-rich internal loop of the PBS structure is important for primer–genome RNA complex stabilization and reverse transcription initiation [42]. Therefore, it may serve as a potential therapeutic target. Since both U-rich RNA structures, the UUU bulge of TAR RNA and the UUUU loop of ASLLys3, play an important role in HIV replication, they may be potential, yet pharmacologically untapped targets for retroviral therapy [31]. Designing new HIV replication inhibitors binding U-rich sequences of viral RNAs creates an opportunity to enhance the arsenal of currently available antiretroviral drugs. NPs of anti-HIV inhibitory properties have been presented in the literature; however, none are currently used in clinical therapy [43,44,45,46]. Because of its unique properties, the Cu(I) ion-catalyzed click reaction (CuAAC) has found widespread use in the synthesis of biologically active compounds, including drugs [47,48,49]. Taking advantage of the success of the click chemistry concept in the design of new drugs, we designed and synthesized a new type of Fmoc-protected nucleo amino acid containing adenine linked to a *L*-azidohomoalanine (Aha) side chain via 1,4-linked-1,2,3-triazole (1,4-TzlNBA_A_). Subsequently, a homotrimer HalTzl_AAA_ complementary to U-rich RNA sequences was synthesized using various strategies of SPPS. The possibility of using a click reaction to attach adenine to the Aha side chain at different stages of the synthesis was tested. Thus, circular dichroism (CD) and fluorescence anisotropy spectroscopy data on the interaction of HalTzl_AAA_ with 5′-fluorescein (FAM(6))-labeled TAR UUU (Fl-TAR UUU) and ASLLys3 (Fl-ASLLys3) structures were presented and discussed.

## 2. Results and Discussion

### 2.1. Synthesis of N9-propargyladenine and Fmoc-1,4-TzlNBA_A_

**Scheme 1:**
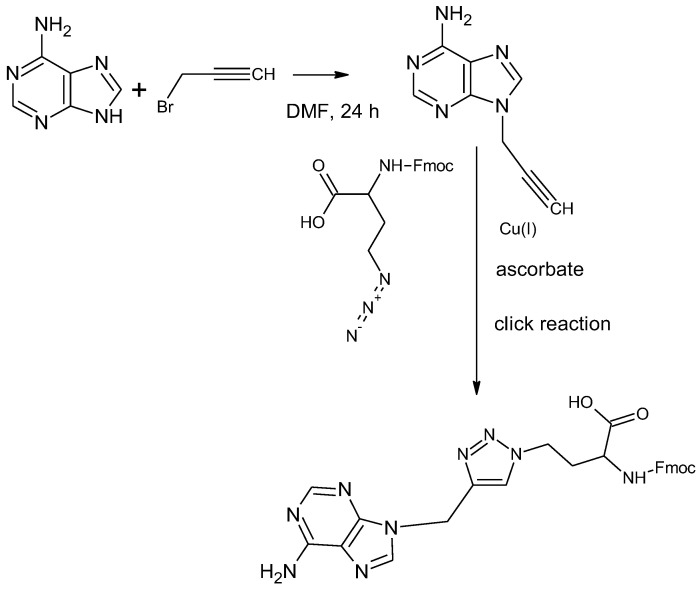
Synthesis of *N9*-propargyladenine and Fmoc-1,4-TzlNBA_A_.

**Scheme 2:**
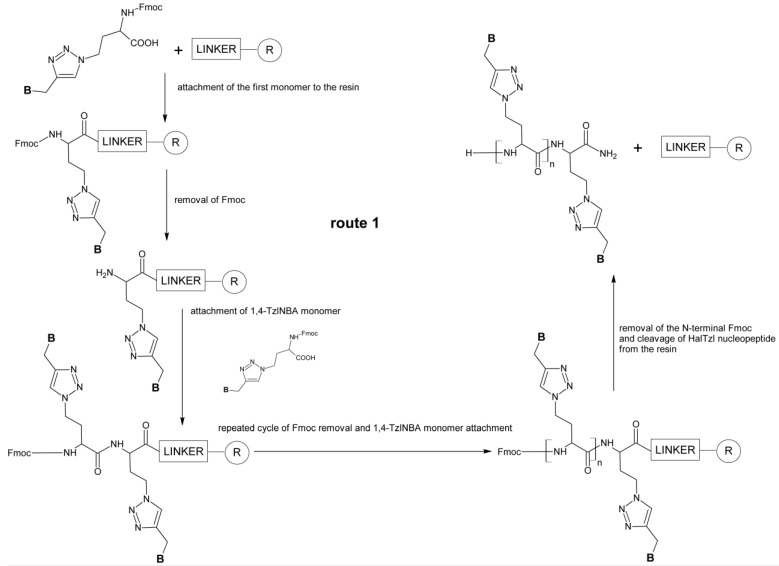
Standard (route 1) solid-phase synthesis of HalTzl_AAA_. Labels: R—resin and B—adenine; n = 2.

In the ongoing pursuit of designing novel NBA monomers to synthesize a new nucleopeptide capable of recognizing unique structures of RNA, we present the synthesis of a novel NBA containing adenine linked through the 1,2,3-triazole ring to the side chain of Fmoc-Aha. The monomer suitable for the SPPS method, Fmoc-1,4-TzlNBA_A_, was synthesized via a Cu(I)-catalyzed click reaction (CuAAC). Unprotected N9-propargyl adenine and commercially available Fmoc-Aha were used as substrates of the click reaction (Scheme 1). Because alkyne derivatives of nucleobases are not commercially available, the alkyne component of the click reaction, N9-propargyladenine, was synthesized using unprotected adenine. It was alkynylated using propargyl bromide at mild basic conditions (potassium carbonate) [11]. The reaction carried out in DMF was regio selective and only N9-izomer (57%, >90% purity) was obtained with a good yield. Following this, the CuAAC reaction was conducted using N9-propargyladenine and Fmoc-Aha with sodium ascorbate as the Cu(II) ion reductant. The click product, Fmoc-1,4-TzlNBA_A_, was obtained with a high yield of 77% (>90% purity) (Table 1). The monomer was conceived as a building block for the HalTzl_AAA_ nucleopeptide, aligning with the Fmoc strategy of SPPS (Scheme 2, route 1).

### 2.2. Solid-Phase Synthesis of HalTzl_AAA_

**Figure 1:**
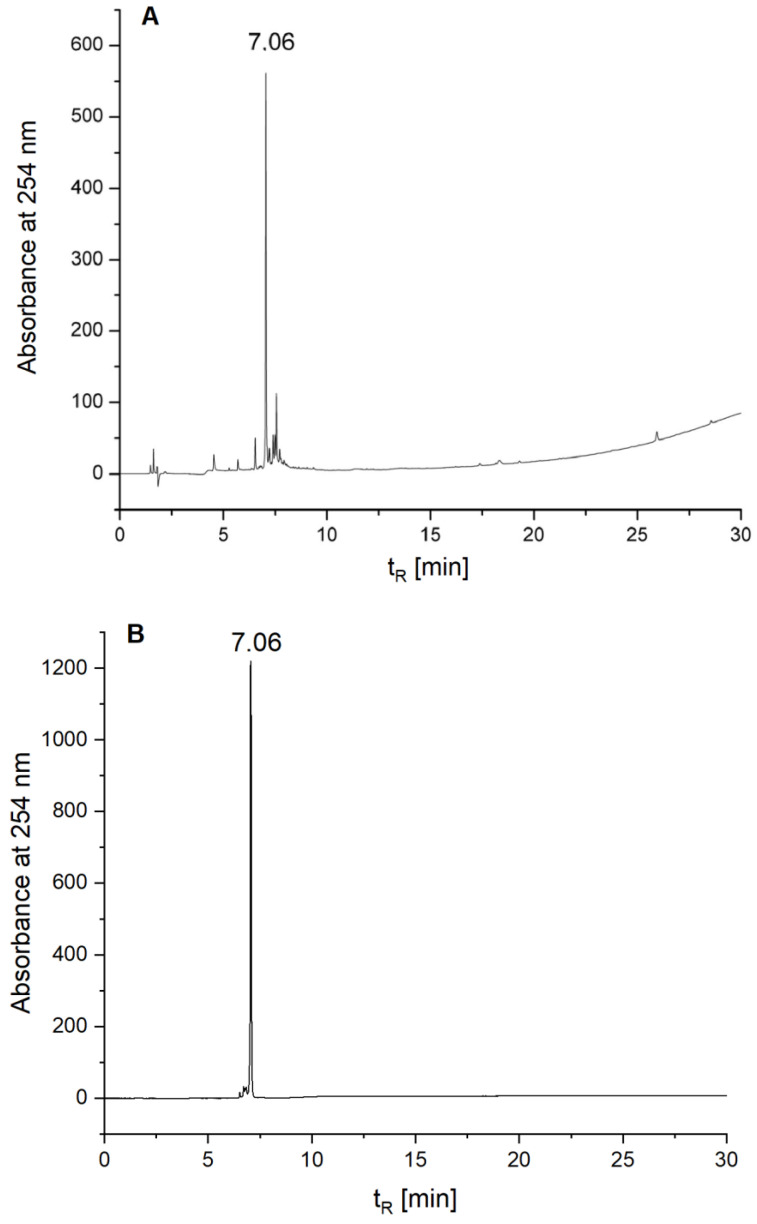
Analytical RP HPLC of crude (**A**) and purified (**B**) HalTzl_AAA_.

**Scheme 3:**
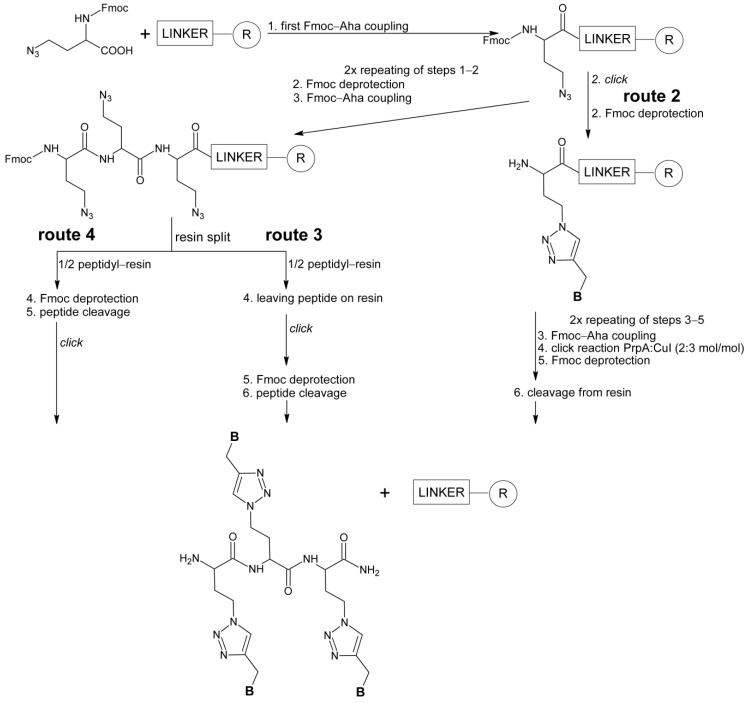
Solid-phase synthesis of HalTzl_AAA_ (route 2–4) with a click reaction. B—adenine. The coupling (A) and Fmoc deprotection (B) conditions: A; the Fmoc-Aha monomer was assembled on the resin in a 3-fold molar excess of coupling reagents (Fmoc-Aha: HATU: HOAt: colidine: DMAP, 1:1:1:2:0.001 molar ratio) in DMF for 60 min. Each coupling was repeated using a 1.5-fold excess of reagents for 60 min. and B; the deprotection was carried out with a 20% piperidine/DMF in two cycles lasting 5 and 15 min, respectively. The reactions proceeded at room temperature.

To characterize the binding of U-rich RNA fragments involved in HIV-1 replication, we synthesized the homotrimer nucleopeptide HalTzl_AAA_ containing a monomeric repeating unit *L*-2-(Fmoc-amino)-4-[4-(N9-methyladenine)-1*H*-1,2,3-triazol-1-yl)butanoic acid (1,4-TzlNBA_A_). HalTzl_AAA_ was synthesized as the C-terminal amide to minimize the unfavorable electrostatic repulsion between the negatively charged phosphate groups of the RNA backbone and the C-terminus of the nucleopeptide. A few different strategies of HalTzl_AAA_ synthesis to explore the possibility of attaching N9-propargyladenine to the azide group-containing side chain of Aha through 1,4-linked 1,2,3-triazole moiety were conducted (Scheme 2 and Scheme 3). The approaches were based on the standard or suitably modified SPPS method. First, the standard protocol for the SPPS method was used, and the Fmoc-1,4-TzlNBA_A_ monomer was assembled on resin (Scheme 1, route 1). Following the synthesis, crude HalTzl_AAA_ nucleopeptide was cleaved from the resin and purified using semi-preparative RP HPLC. A representative RP HPLC chromatogram of a crude HalTzl_AAA_ is shown in Figure 1. The analysis showed that a product of over 70% purity was obtained after the synthesis. A similar result was obtained using modified versions of SPPS (Scheme 3, routes 2–4). An analytical RP HPLC chromatogram confirmed the homogeneity of the final product (Figure 1). The second strategy of the HalTzl_AAA_ synthesis was based on a click reaction between N9-propargyladenine and Aha previously attached or cleaved from the resin (Scheme 3, routes 2–4). The synthesis was carried out in two variants. In the first one, after the attaching of Fmoc-Aha to the resin, N9-propargyladenine was coupled via a click reaction to the azide group located in the side chain of the attached amino acid (Scheme 3, route 2). A 1,2-fold molar excess of the adenine derivative relative to Aha was used. The progress of the click reaction was not monitored. The entire cycle of attaching Aha to the resin and the following attaching/clicking of N9-propargyladenine was repeated three times during the synthesis. Thus, HalTzl_AAA_ nucleopeptide was cleaved from the resin, purified and characterized.

In the second variant, using the standard SPPS protocol, the (Aha)_3_-resin trimer was synthesized on resin (Scheme 3). Then, the peptidyl resin was divided in half. To one part, N9-propargyladenine was attached/clicked to the azide groups of the three Aha residues attached to the resin (Scheme 3, route 3). Here, too, the progress of the reaction was not monitored. After synthesis, the HalTzl_AAA_ nucleopeptide was cleaved from the resin, purified and characterized. After the removal of the N-terminal Fmoc group, a tripeptide (Aha)_3_-amide was cleaved from the second portion of the resin, and then N9-propargyladenine was clicked to the azide groups located in the side chains of the three Aha residues (Scheme 3, route 4). The nucleopeptide HalTzl_AAA_ thus obtained was then subjected to purification and characterized. Different approaches to the HalTzl_AAA_ synthesis had similar effectiveness (Table 1). Regardless of the synthesis path chosen, the crude product with a similar efficiency and purity was obtained.

### 2.3. CD Studies of RNAs–HalTzl_AAA_ Interaction

**Figure 2:**
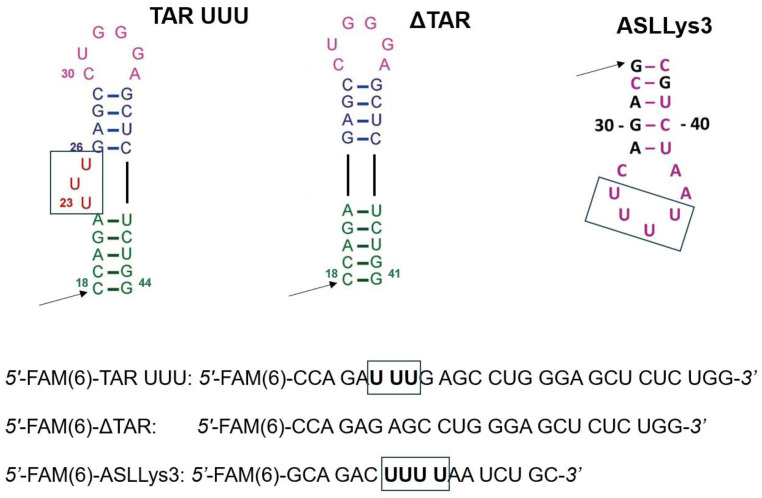
Secondary structures (unlabeled) and sequences (bottom, labeled) of TAR UUU, ΔTAR, and ASLLys3 being the targets for HalTzl_AAA_ are boxed. Arrows show the place of FAM(6) labeling. U-rich sequences are boxed.

To characterize the ability of HalTzl_AAA_ to bind U-rich RNA structures important in the HIV-1 replication cycle, circular dichroism (CD) and fluorescence spectroscopy were used. Due to their potential pharmacological significance, two fragments of RNAs involved in HIV replication were selected, a 27-nucleotide fragment of the transactivation response element (TAR) responsible for the elongation of viral mRNAs and a 17-nucleotide fragment of the unmodified anticodon stem-and-loop domain of human tRNALys3 (ASLLys3), responsible for the priming and initiation of reverse transcription of the genomic viral RNA [38]. In the case of the TAR RNA structure, a mutant containing the UUU bulge was selected, and the bulge was selected as a target for HalTzl_AAA_. In the case of ASLLys3, its UUUU fragment located in the outer loop was chosen as a target sequence (Figure 2).

For CD experiments, unlabeled versions of TAR UUU and ASLLys3 were used, whereas for fluorescence studies, its 5′-labeled with the fluorescein (FAM) 6-isomer was used. (FAM(6)) analogs labeled as 5′-FAM(6)-TAR UUU and 5′-FAM(6)-ASLLys3 were applied (Figure 2).

#### CD Study of TAR UUU RNA–HalTzl_AAA_ Interaction

**Figure 3:**
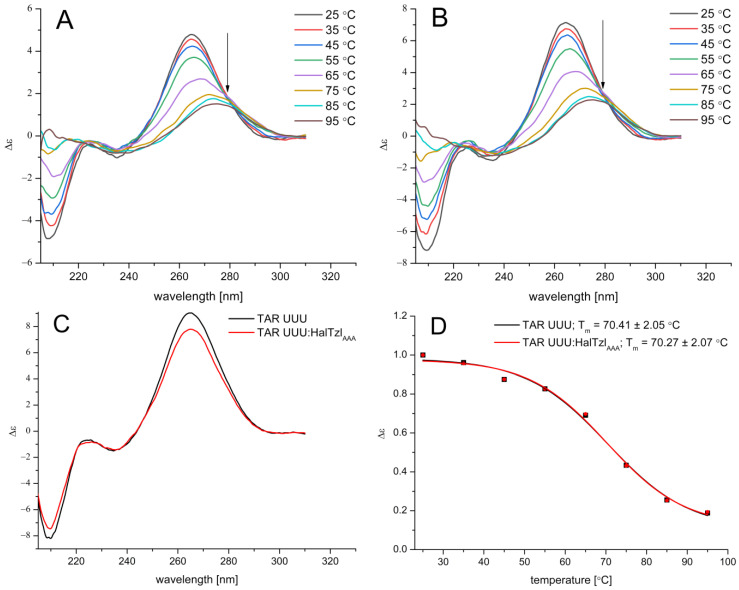
Temperature-dependent CD spectra for free TAR UUU (**A**) and TAR UUU–HalTzl_AAA_ (**B**). CD spectra of the TAR UUU–HalTzl_AAA_ interaction (**C**) and CD melting curves of free TAR UUU and the TAR UUU–HalTzl_AAA_ complex (**D**). A black arrow shows a progressive increase in temperature.

Temperature-dependent CD studies were employed to characterize both uncomplexed and bound TAR UUU. CD spectra of form A RNA typically exhibit a negative minimum around 210 and 235 nm and a positive maximum at 265 nm [5,11]. The latter serves as a diagnostic band for ligand binding, as its intensity and position are sensitive to changes in the nucleobase stacking profile. These changes correlate with conformational alterations in RNA induced by physicochemical factors (e.g., temperature) or ligand binding. The CD spectrum of free TAR UUU recorded is characteristic of the A form of RNA (Figure 3). The spectra exhibit three extremes: a minimum at about 208 nm and 235 nm and a maximum at 265 nm. To assess the stability of the TAR UUU hairpin–bulge structure, temperature-dependent CD measurements were conducted. CD curves recorded in the range of 25–95 °C showed that the structure of TAR UUU was strongly temperature-dependent (Figure 3). A progressive decrease in the intensity of both extremes correlated with increasing temperature. In the case of the maximum at 265 nm, an additional shift toward longer wavelengths was observed. These changes reflect conformational changes in single-stranded RNA fragments, the bulge, and the apical loop, as well as the unraveling of double-stranded helix fragments located both above and below the bulge. Temperature-dependent changes in ellipticity at the maximum of around 265 nm were used to characterize the stability of unbound and complexed TAR UUU structures by determining their melting temperatures (T_m_). The free TAR UUU CD melting profile is shown in Figure 3A. The curves were characterized by the distinctive sigmoid-like shape typical of unraveling double-stranded TAR fragments. The determined T_m_ value indicated that the structure of TAR UUU is stable in TB buffer (T_m_ 70.41 ± 2.05) (Table 2, Figure 3D). A similar value was previously reported in the literature [50]. Subsequently, CD was employed to characterize the binding of HalTzl_AAA_ to the TAR UUU (Figure 3B,C). As the quantitative characterization of the target interactions was determined by dissociation constants (K_d_) using fluorescence anisotropy spectroscopy, CD experiments focused on the analysis of complexes formed under conditions of a 10-fold excess of HalTzl_AAA_ ligands relative to TAR. CD curves illustrating the interaction of TAR UUU with HalTzl_AAA_ ligands are characterized by a reduction in the intensity of the band ellipticity at 265 nm and a less intense effect for the band at 210 nm (Figure 3C). Since HalTzl_AAA_ themselves exhibits a negative ellipticity value in the 200–240 nm range, the CD curves of the complexes were corrected for this effect.

The decrease in ellipticity intensity for the band at approximately 265 nm was approximately 15%. With increasing temperature, CD melting curves were characterized by a gradual decrease in the intensity of the CD signal at a maximum of 265 nm and a shift in this maximum toward longer wavelengths (Figure 3B). At the highest temperature investigated (95 °C), the CD maximum was moved to about 275 nm. These changes were similar to those observed for unbound TAR UUU (Figure 3A). CD melting curves show that the stability of unbound and complexed TAR UUU, characterized by the T_m_ value, are almost the same (Figure 3D, Table 2).

**Figure 4:**
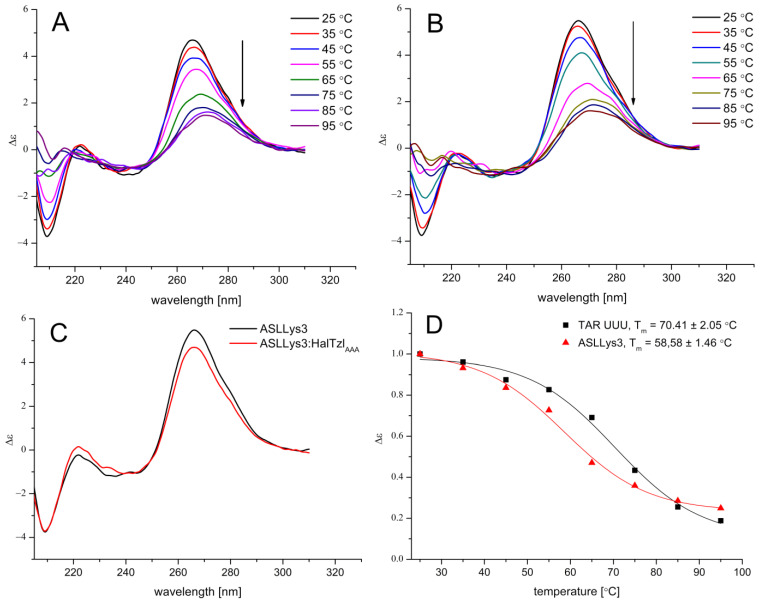
Temperature-dependent CD spectra for free ASLys3 (**A**) and the ASLLys3–HalTzl_AAA_ interaction (**B**). CD spectra of the ASLLys3–HalTzl_AAA_ interaction (**C**) and normalized CD melting curves for free and bound ASLLys3 (**D**). A black arrow shows a progressive increase in temperature.

These may indicate a moderate affinity of HalTzl_AAA_ for the TAR UUU bulge. CD melting curves (Figure 3D) show virtually no change in T_m_ values for free and complexed TAR UUU. This suggests that HalTzl_AAA_ binding may occur in the U-rich stem region, while the duplex regions above and below the stem remain unaffected. This would be confirmed by the decrease in the intensity of the ellipticity band at 260 nm observed after the addition of the ligand to TAR UUU (Figure 3C). Usually, such an effect is correlated with the rearrangement of nucleobase associations in single-stranded RNA regions, in this case the bulge. The combination of observations of changes in the intensity of the ellipticity band at 260 nm (Figure 3C) with the practically unchanged Tm value observed for free and complexed TAR UUU (Figure 3D) suggests that HalTzl_AAA_ binding causes only local (probably in the stem region) and not global changes in the structure of the RNA fragment. For the ASLLys3 fragment, which is the unmodified domain of the htRNALys3 anticodon domain, a similar temperature dependence of the ellipticity signal was observed as for TAR UUU (Figure 4A). At 25 °C, the CD curve exhibits a typical characteristic A form RNA with an ellipticity maximum at 266 nm and a minimum at 208 nm. As the temperature increases, a gradual decrease in the intensity of the CD extremes is observed, coupled with a shift in the 266 nm maximum toward longer wavelengths to about 270 nm. Furthermore, the analysis of CD melting curves recorded for changes in the CD maximum at 266 nm indicates that the ASLLys3 structure is much less stable (Tm~58.6 °C) compared to the TAR UUU structure (Figure 3A, Table 2). This is probably due to smaller number of double-stranded fragments in the ASLLys3 structure than in the TAR UUU. The temperature-dependent characteristics of the CD curves recorded for ASLLys3 complexed with HalTzl_AAA_ are similar to those recorded for unbound ASLLys3 (Figure 4A,B). During the complexation, the intensity of the maximum at 265 nm decreased and slightly increased at the maximum at 220 nm (Figure 4C). The latest effect was not observed for the TAR UUU–HalTzl_AAA_ interaction. An analysis of CD melting curves showed that the complexation with HalTzl_AAA_ slightly enhanced the stability of the ASLLys3 structure. The CD melting curves for both unbound and complexed ASLLys3 are similar to each other. A slight difference in T_m_ values of approximately 0.5 °C shows that the ligand binds to the U-rich sequence of the outer loop of ASLLys3 with moderate affinity (Figure 4D, Table 2).

### 2.4. Fluorescence Anisotropy Study of TAR RNA–HalTzl_AAA_ Interactions

**Figure 5:**
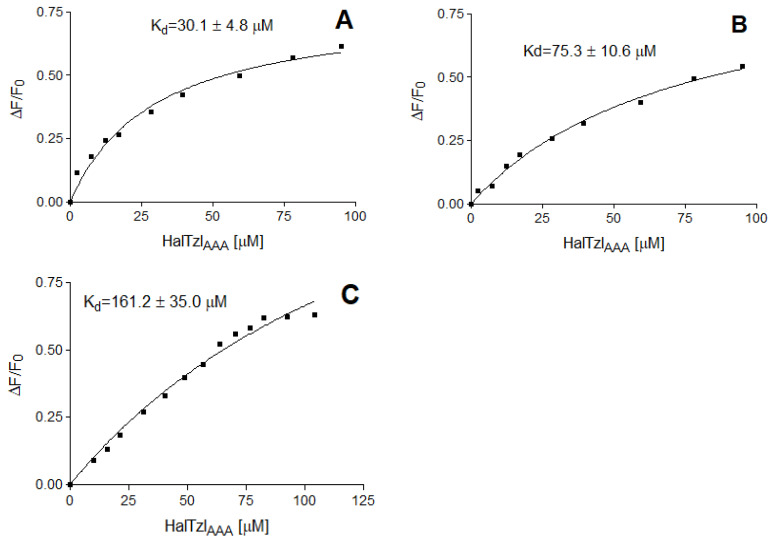
Fluorescence anisotropy binding curves obtained by titration of Fl-TAR UUU (**A**), Fl-ASLLys3 (**B**), and ΔTAR (**C**) with HalTzl_AAA_. Details are shown in Materials and Methods, Section 3.6.

Fluorescence anisotropy was employed to quantify RNAs–HalTzl_AAA_ interactions. Fluorescently labeled RNAs, 5′-FAM(6)-TAR UUU (labeled as Fl-TAR UUU) and nd 5′-FAM(6)-ASLLys3 (labeled as Fl-ASLLys3), were utilized as target molecules to characterize the binding properties of the HalTzl_AAA_ ligand. The binding experiments involved measuring the decreasing fluorescence anisotropy of fluorescently labeled RNAs caused by the addition of increasing amounts of the ligand (Figure 5).

The obtained data were used to determine binding curves. The dissociation constants (K_d_) of the Fl-TAR UUU–HalTzl_AAA_ complex was then calculated (Table 3). To derive the binding curves and K_d_ values, it was assumed that the interaction corresponds to the one-binding-site model, where the HalTzl_AAA_ ligand binds to the complementary sequences of the bulge or the apical loop regions of TAR UUU and ASLLys3, respectively.

The analysis of TAR UUU interactions with HalTzl_AAA_ revealed a medium-strength binding characterized by a dissociation constant (K_d_) of approximately 30 µM (Figure 5A, Table 3). The binding constant of the ASLLys3–HalTzl_AAA_ interaction was determined to be around 75 µM (Figure 5B). This value indicates the moderate affinity of the ligand for the complementary UUUU sequence present in the anticodon loop. This value is almost twofold higher than that observed as a result of interaction with the UUU bulge of the TAR structure, thereby confirming that Watson–Crick-type complementarity is not the only affinity determinant. In both cases, the target complementary sequence consists exclusively of uridine residues. Consequently, it appears that the determining factor for affinity in this case is the conformational differences of the target complementary RNA fragments.

A TAR mutant, lacking the U-rich bulge sequence (designated as ΔTAR) and thus unable to bind HIV-1 Tat protein, was used as a negative control for HalTzl_AAA_ binding specificity. ΔTAR has a hairpin structure resembling that of ASLLys3. Both structures have external loops of similar sizes (six vs. seven nucleotides) (Figure 2). Although the stem length is different (9 vs. 5 bp), in both cases, these structures are rich in GC pairs (Figure 2). 5′-FAM(6)-labeled ΔTAR (labeled as Fl-ΔTAR) showed low affinity for HalTzl_AAA_ (K_d_~161 µM) (Table 3, Figure 5C). This value is more than two to five times higher than in the cases of ASLLys3 and TAR UUU, respectively (Table 3). This shows that U-rich RNAs are recognized with significantly higher affinity than those lacking these motifs, leading to the conclusion that U-rich motifs are an important determinant of the binding specificity of the A-rich nucleopeptide HalTzl_AAA_.

The HalTzl_AAA_ ligand demonstrated moderate (approximately 30–75 µM) affinity for the TAR UUU and ASLLys3 structures. This implies that interactions, such as hydrogen bonding or hydrophobic interactions involving complementary nucleobases and triazole rings, are insufficient to yield ligands with high affinity. It is likely that introducing positively charged amino acid residues, such as lysine (Lys) or arginine (Arg), into a newly designed structure of HalTzl_AAA_ would be a reasonable strategy to increase both the specificity and affinity of U-rich structure recognition. This approach, commonly used in RNA ligand design, involves additional electrostatic interactions between the positively charged side chains of Lys or Arg and the negatively charged phosphate groups of the sugar–phosphate chain of RNA.

The popularity of click reaction, especially the CuAAC variant, and the ease of formation of the triazole ring have made the design of a new triazole containing molecules an important branch of synthetic organic and medicinal chemistry [51]. Many examples show that the newly designed triazole-based derivatives of known molecules had stronger biological activity than their unmodified leading counterparts [51,52,53]. The molecular basis of such modifications is poorly understood. Previous results have shown the influence of triazole-modified 2′-deoxyuridines on the stability of nucleic acid duplexes [54,55]. It was observed that although the incorporation of a single triazole ring decreased DNA/DNA duplex stability, the stacking of a few (2–4 triazole residues) consecutive modifications led to increased stability due to the additional base stacking interactions of triazole moieties. This demonstrates the complex nature of the interactions of the triazole system with the structure of the nucleic acid chain, both nucleobases and the backbone, which are likely to involve electrostatic and hydrophobic interactions, including base stacking, the possibility of forming hydrogen bonds, as well as steric hindrance, as the triazole ring is a flat aromatic system similar in size to the pyrimidine nucleobase. The presented results concerning various HalTzl_AAA_ synthesis strategies show that the triazole ring enabling the incorporation of a nucleobase into the amino acid side chain can be effectively formed using a click reaction during synthesis on resin, as well as after peptide cleavage. This broadens the range of possibilities for the design and synthesis of compounds containing a triazole system using the solid-phase synthesis method. Our strategies of linking adenine to the side chain of Aha through the triazole ring is a small step towards understanding the influence of the triazole ring on the process of RNA recognition by such modified NPs.

## 3. Materials and Methods

All reagents were purchased from commercial suppliers without additional purification. Fmoc-*L*-Aha (N-α-(9-Fluorenylmethyloxycarbonyl)-4-azido-*L*-homoalanine) and other reagents suitable for SPPS were purchased from Iris Biotech GmbH (Marktredwitz, Germany), and adenine and propargyl bromide were obtained from Merck Life Science (Poznan, Poland). RinkAmide TentaGel S RAM resin was purchased from Rapp Polymere GmbH (Tuebingen, Germany). Simple organic and inorganic chemicals like solvents or organic/inorganic salts were purchased from Merck Life Science (Poznan, Poland). Aqueous solutions were freshly prepared using distilled deionized water from a Milli-Q Millipore system (Bedford, MA, USA) and filtered through a 0.22 µm PTFE filter before use. The progression of the reactions was monitored using TLC with Merck precoated aluminum plates 60 F_254_ (Merck Life Science, Poznan, Poland) featuring a 0.2 mm layer of silica gel containing a fluorescence indicator. Analytical RP HPLC analyses were conducted on a Beckman instrument (Beckman Instruments, Fullerton, CA, USA) using a Phenomenex Kinetex C18 column (4.6 mm × 150 mm, 5 µm particle size, Phenomenex, Torrance, CA, USA). Flash chromatography or preparative RP HPLC purifications were performed on a silica gel column (DCM:MeOH, 95:5 *v*/*v*) or a Spot Prep II 250 instrument (Armen Instrument, Saint-Ave, France) utilizing a C18 reversed-phase column. The ^1^H and ^13^C NMR measurements were carried out in DMSO-d_6_ using a Bruker AVANCE III 500 MHz spectrometer (Bruker Daltonics, Billerica, MA, USA) and standard Bruker software (Analyst TF 1.7.1.); δ in parts per million; J in hertz. Mass spectra were recorded on a triple quadrupole time-of-flight (QTOF) mass spectrometer AB SCIEX 5600+ (AB Sciex LLC, Framingham, MA, USA) operated in a positive ionization mode.

### 3.1. Synthesis of N9-Propargyladenine

*N9*-propargyladenine was obtained by reacting unprotected adenine with propargyl bromide in the presence of potassium carbonate in DMF at room temperature, as described in the literature [11]. A mixture of 270 mg (2 mmol) of adenine, 263 mg (1.9 mmol) of K_2_CO_3_, and 152 µL/238 mg (2 mmol) of propargyl bromide in 20 mL of anhydrous DMF (dried over molecular sieves A4) was stirred at room temperature for 24 h. After removing the solvent under reduced pressure, the residue obtained was purified on a silica gel column (DCM:MeOH, 95:5 *v*/*v*). *N9*-propargyladenine was obtained as a white solid (98.71 mg, 57%). The reaction was scalable up to 10 times the amount of the reactants. ESI-MS: *m*/*z* calcd and found for [M + H]^+^ 174.070. The [M + K]^+^ 212.086 ion was also found.

### 3.2. Synthesis of L-2-(Fmoc-amino)-4-[4-(N9-methyladenine)-1H-1,2,3-triazol-1-yl)butanoic Acid (1,4-TzlNBA_A_)

To a mixture of 173 mg (1 mmol) of *N9*-propargyladenine and 440 mg (1.2 mmol) of Fmoc-Aha dissolved in H_2_O/*t*-BuOH (1:1 *v*/*v*), a 100 mM solution of CuSO_4_ × 5H_2_O (1.2 mmol) and a freshly prepared 500 mM solution of sodium ascorbate (4 equiv) were added. The reaction mixture was stirred at room temperature for 1 day and monitored by RP HPLC. After completion, the resulting slurry containing synthesized *L*-2-(Fmoc-amino)-4-[4-(*N9*-methyladenine)-*1H*-1,2,3-triazol-1-yl)butanoic acid (Fmoc-1,4-TzlNBA_A_) was filtered, and the solvent was removed under reduced pressure. The crude product was pre-purified by precipitation from DMSO with cold water. A preparative C18 RP HPLC for the final purification was then applied. Finally, a white solid (415.45 mg, 77%) of 1,4-TzlNBA_A_ was obtained. ESI-MS: *m*/*z* calcd for [M + H]^+^ 540.210; found: 540.157.

^1^H NMR (DMSO-d6, δ): 12.81 (1H, COOH, s); 8.26 i 8.21 (2H, H2 i H8, 2s); 8.09 (1H, H5(Tzl), s); 7.90 (2H, Fmoc, d, J = 7.4 Hz); 7.79 (1H, NH(Hal), d, J = 8.15 Hz); 7.73 (2H, Fmoc, d, J = 5.5 Hz); 7.61 (2H, NH_2_, s); 7.42 (2H, Fmoc, ddd, J = 4.15 Hz); 7.33 (2H, Fmoc, dd, J = 6.4 Hz); 5.46 (2H, Tzl-CH_2_-A, s); 4.38 (2H, Tzl-***CH*_2_**-CH_2_, m); 4.33 (2H, OCH_2_, d, J = Hz); 4.25 (1H, CH(Fmoc), t, J = 6.9 Hz); 3.91 (1H, CH(Hal), m); 2.30 i 2.11 (2H, Tzl-CH_2_-***CH*_2_**, m). ^13^C NMR (DMSO-d6, δ): 173.49; 156.60; 151.60; 149.60; 144.26; 142.84; 141.21; 128.12; 127.56; 125.70; 124.17; 120.61; 66.09; 51.63; 47.06; 38.64; 31.67.

### 3.3. Solid-Phase Synthesis of HalTzl_AAA_ Nucleopeptide

#### 3.3.1. Standard SPPS of HalTzl_AAA_ (Route 1)

Nucleopeptide HalTzl_AAA_ was synthesized as a C-terminal amide using a semi-automatic peptide synthesizer, Peptide Synthesizer SP650 Labortec AG (Bubendorf, Switzerland). A few different strategies of SPPS were applied. A classic one was based on assembling the Fmoc-1,4-TzlNBA_A_ monomer on resin (Scheme 1, route 1). The second one was based on a click reaction between N9-propargyladenine and Aha attached to the resin (Scheme 2, routes 2 and 3). A variant of attaching N9-propargyladenine via a click reaction to the nuleopeptide cleaved from the resin was also tested (Scheme 2, route 4).

In the first case, the classical SPPS method with the Fmoc strategy was used [11]. The Fmoc-1,4-TzlNBA_A_ monomer was assembled on TentaGel S RAM resin (capacity 0.22 mmol Fmoc-NH/1 g resin; 50 mg resin was used) as active derivatives in a 3-fold molar excess of coupling reagents (Fmoc-1,4-TzlNBA_A_: HATU: HOAt: colidine: DMAP, 1:1:1:2:0.001 molar ratio) in the *N*,*N*-dimethylformamide (DMF) solution for 60 min. Each coupling process was repeated using a 1.5-fold excess of reagents for 60 min. The removal of the Fmoc groups was carried out with a 20% piperidine/DMF in two cycles lasting 5 and 15 min, respectively. Once the reaction was complete, the resin was washed with dichloromethane and dried in a vacuum desiccator. Subsequently, the HalTzl_AAA_ nucleopeptide was cleaved from the resin and deprotected with a 98% TFA/DCM solution for 2 h at room temperature under an inert gas (argon). The nucleopeptide was then precipitated with cold diethyl ether, filtered, dissolved in water, frozen, and lyophilized. Crude HalTzl_AAA_ were purified using a semi-preparative RP HPLC Knauer chromatograph (Knauer, WG GmbH, Berlin, Germany) system using a Kromasil C8 column (20 × 250 mm, 10 µm particle size). A gradient of 0–60% ACN with 0.08% TFA, at a flow rate of 3.5 mL/min, was used for purification. The eluates were monitored with a UV detector at *λ* = 254 nm. Fractions with the highest purity (>95%) were analyzed by analytical RP-HPLC using a Beckman System Gold chromatograph (Beckman Coulter, Brea, CA, USA) using a Kinetex C18 column (Phenomenex, 150 × 4.6 mm, 5 µm particle size) with a 0–100% ACN gradient with the addition of 0.08% TFA (solvent B) and 0.08% TFA (solvent A) in 30 min. The column was maintained at ambient temperature. The flow rate was 1 mL/min, and the eluates were monitored using a UV detector at *λ* = 254 nm. Finally, after purification and lyophilization, 4.2 mg (36.7% yield, >95% purity) of HalTzl_AAA_ was obtained. The identity of HalTzl_AAA_ was confirmed by mass spectrometry using a QTOF SCIEX 5600+ ESI spectrometer operating in positive mode. A summary of the physical and chemical parameters of HalTzl_AAA_ can be found in Table 1.

#### 3.3.2. Solid-Phase Synthesis of HalTzl_AAA_ with a Single 1,4-TzlNBA_A_ Residue Click on Resin (Route 2)

Initially, the synthesis (Scheme 2, route 2) was carried out analogously to that described in Section 3.3.1, except that Fmoc-Aha was attached to the resin. A total of 20 mg of resin (44 µmoles scale) was used. The single cycle of Fmoc-Aha attachment to the resin was extended by an additional step of attaching N9-propargyladenine to the azide group-containing side chain of the freshly attached Fmoc-Aha residue via a click reaction. A mixture of N9-propynyladenine:CuI (2:3 molar ratio relative to the number of azide groups of freshly attached Fmoc-Aha) was dissolved in a solution of DMF: DIPEA (4:1 *v*/*v*). The reaction was carried out for 24 h without light at room temperature. The progress of the click reaction was not monitored. The resin was then drained and washed three times with DMF and DCM. Finally, the Fmoc group was removed, and then another cycle of amino acid attachment to the resin was started. The Fmoc group was removed before the nucleopeptide cleavage. After completion of the synthesis, the HalTzl_AAA_ nucleopeptide was cleaved from resin, precipitated from the cleavage mixture, washed, redissolved, frozen, and lyophilized according to the procedures described in Section 3.3.1. After purification, 0.6 mg (30% yield, >95% purity) of HalTzl_AAA_ was obtained. The identity of the final product was confirmed by MS ESI.

#### 3.3.3. Solid-Phase Synthesis of HalTzl_AAA_ with a Simultaneous All 1,4-TzlNBA_A_ Residues Click on Resin (Route 3)

Initially, the synthesis (Scheme 2, route 3) was carried out analogously to that described in Section 3.3.1, except that Fmoc-Aha instead of Fmoc-1,4-TzlNBA_A_ was attached to the resin. A total of 20 mg of resin (44 µmoles scale) was used. The single cycle of Fmoc-Aha attachment to the resin was accomplished using the protocol described in Section 3.3.1. After the attachment of the three Aha residues, a click reaction was performed on the resin according to the protocol described in Section 3.3.2. The click reaction was carried out for 24 h without light at room temperature. The progress of the click reaction was not controlled. After the completion of the synthesis and removal of the N-terminal Fmoc group, the HalTzl_AAA_ nucleopeptide was cleaved from resin. Crude HalTzl_AAA_ was purified according to the procedure described in Section 3.3.1. Finally, 0.52 mg (26% yield, >95% purity) of HalTzl_AAA_ was obtained.

#### 3.3.4. Post-Cleavage Click Synthesis of HalTzl_AAA_ (Route 4)

Initially, the synthesis (route 3) was carried out analogously to that described in Section 3.3.1, except that Fmoc-Aha was attached to 20 mg (44 µmoles scale) of the resin. After completion of the synthesis and removal of the N-terminal Fmoc group, the (Aha)_3_-amide tripeptide was cleaved from the resin and lyophilized according to the procedure described in Section 3.3.1. Then, the crude peptide was dissolved in a minimal amount of water, followed by the addition of a mixture of N9-propargyladenine:CuI:TBTA (4:0.005:0.01 molar ratio relative to the number of azide groups in the peptide) dissolved in 50% DMF. The reaction was carried out for 24 h without light at room temperature. The progress of the click reaction was controlled by analytical RP HPLC. The synthesized crude HalTzl_AAA_ were purified using a semi-preparative RP HPLC and characterized as described in Section 3.3.1. Finally, 0.6 mg (30% yield, >95% purity) of HalTzl_AAA_ was obtained.

### 3.4. U-Rich RNAs Synthesis

5′-FAM(6)-TAR UUU (labeled as Fl-TAR UUU), 5′-FAM(6)-ASLLys3 (labeled as Fl-ASLLys3), 5′-FAM(6)-ΔTAR (labeled as Fl-ΔTAR), and its unlabeled forms (except for ΔTAR) were obtained from FutureSynthesis (Poznan, Poland). The lyophilized samples were dissolved in TB buffer and denatured at 95 °C for 5 min before CD and fluorescence anisotropy experiments.

### 3.5. Circular Dichroism (CD) Spectroscopy

CD spectra were recorded using a Jasco J-815 spectropolarimeter (Jasco, Tokyo, Japan) equipped with a Peltier-controlled cell holder in a quartz cuvette with a 0.1 cm path length spanning from 200 to 310 nm at 25 °C. CD temperature-dependent experiments were conducted over the range of 25–95 °C. TAR UUU RNA was maintained at a concentration of 5 μM for all CD experiments. To prevent changes in ellipticity values linked to potential sample dilution, the solution containing TAR UUU RNA was introduced to the dry lyophilized HalTzl_AAA_ sample, resulting in a final TAR UUU RNA concentration of 5 μM and a 10-fold excess (50 μM) of nucleopeptide concentration. Before initiating CD experiments, each sample was equilibrated for at least 30 min at the initial temperature to ensure the required temperature and achieve equilibrium in the TAR UUU–HalTzl_AAA_ interaction. The spectra were averaged over three scans, each recorded at a speed of 100 nm/min with a 1 nm data pitch. As the HalTzl_AAA_ nucleopeptide exhibits a negative ellipticity effect in the 200–240 nm range, ellipticity values for the TAR UUU–HalTzl_AAA_ complex was corrected for the contribution associated with HalTzl_AAA_. The observed ellipticity for both free and bound (corrected ellipticity) TAR was expressed in mdeg. The baseline was corrected for every CD spectrum. Origin 6.5 software (OriginLab, Northampton, MA, USA) was utilized for the analysis of obtained data in the CD melting experiments. The Boltzmann function implemented in this program was employed for fitting melting curves and calculating T_m_ values.

### 3.6. Fluorescence Anisotropy Spectroscopy

The binding affinities between HalTzl_AAA_ and Fl-TAR UUU, Fl-ASLLys3, and Fl-ΔTAR structures were determined through fluorescence anisotropy measurements. This was conducted using a Fluoromax-4 spectrofluorophotometer (Horiba Jobin Yvon IBH Ltd., Kyoto, Japan) with a cylindrical quartz cuvette featuring an optical path of 3 mm. Fluorescence anisotropy was computed from the intensities detected at 518 nm with excitation at 480 nm. Each data point was measured three times with a 500 ms integration time and then averaged. Fl-TAR UUU or Fl-ASLLys3 solution (final concentration 100 nM) underwent titration with increasing amounts of HalTzl_AAA_. The complexation was performed in TB buffer at 25 °C. HalTzl_AAA_ (ligand) solutions were prepared as serial dilutions in TB buffer. Before initiating the experiment, the FAM-labeled TAR RNA/ASLLys3 samples were refolded by heating to 90 °C for 5 min and then being allowed to cool freely to 25 °C. Subsequently, the titration was performed. After adding the ligand to the RNA, the sample was gently mixed by hand and incubated to achieve complexation equilibrium at 25 °C for 20 min. Binding data were analyzed using a single binding site curve-fitting procedure implemented in GraphPad Prism 3.0 software (GraphPad Software Inc., San Diego, CA, USA). The equilibrium dissociation constants (K_d_s) for each fluorescence experiment were assessed using the one-site hyperbolic binding equation ΔF/F_0_ = B_max_[C]/(K_d_ + [C]). The fluorescence quenching efficiency was defined as ΔF/F_0_ = (F_0_ − F)/F_0_, where F and F_0_ are the fluorescence anisotropy intensities of Fl-RNAs solutions with and without HalTzl_AAA_, respectively. B_max_ is the maximum fluorescence quenching intensity when the binding is saturated.

## 4. Conclusions

In summary, an Fmoc-protected nucleobase 1,2,3-triazole-linked amino acid derivative (1,4-TzlNBA_A_) has been synthesized using click chemistry (CuAAC). The monomer was suitable for the solid-phase synthesis of a new type of nucleopeptide, HalTzl_AAA_. Both the classic SPPS method and its modifications, which take into account the click reaction carried out both during nucleopeptide synthesis on the resin and after its cleavage, have proven effective in the synthesis of the new class of nucleopeptides containing adenine attached to the homoalanine side chain via a triazole ring. Interaction studies using CD and fluorescence anisotropy have demonstrated that the designed HalTzl_AAA_ ligands bind to complementary U-rich sequences of TAR UUU and ASLLys3, albeit with moderate affinity. This suggests that introducing additional positively charged basic amino acid residues, such as Lys and Arg, into the chain of HalTzl_AAA_ could, through extra electrostatic interactions, enhance the affinity of the ligand. The more than 5-fold-higher affinity of HalTzl_AAA_ for TAR UUU compared to ΔTAR (a mutant lacking the UUU bulge) shows that the U-rich motif is an important element of ligand binding specificity for RNA. The presented results may contribute to the development of new inhibitors blocking TAR-dependent transactivation and tRNALys3-priming reverse transcription during HIV replication and a better understanding of the mechanisms governing the recognition and control of U-rich RNA motifs.

## Figures and Tables

**Table 1:** Summary of the physicochemical parameters of Fmoc-1,4-TzlNBA_A_ and HalTzl_AAA_.

Monomer/Nucleo- Peptide Label	[M + H]^+^		RPHPLC	Quantity Yield % (mg)	
	Calculated	Determined	Analytical RP HPLC Gradient	t_R_ [min]	
Fmoc-1,4-TzlNBA_A_	540.21	540.16	0–100%B in 30 min	14.55	77% (415.45)
HalTzl_AAA_	915.42	915.42 *	0–100%B in 30 min	7.06	route 1–37% (4.20) route 2–30% (0.60) route 3–26% (0.52) route 4–29% (1.16)

* [M + 2H]^2+^ = 458.209 and [M + 3H]^3+^ = 305.807 ions were also detected.

**Table 2:** Summary of melting temperatures (T_m_) determined from CD melting curves.

Free/Complexed TAR/ASL	T_m_ [°C]	ΔT_m_ [°C]
TAR UUU	70.41 ± 2.05	-
TAR UUU—HalTzl_AAA_	70.27 ± 2.07	−0.14
ASLLys3	58.58 ± 1.46	-
ASLLys3—HalTzl_AAA_	59.20 ± 0.89	0.62

**Table 3:** Dissociation constants (K_d_) of the Fl-RNAs–HalTzl_AAA_ interaction.

Complex	K_d_ [µM]
Fl-TAR UUU–HalTzl_AAA_	30.1 ± 4.8
Fl-ASLLys3–HalTzl_AAA_	75.3 ± 10.6
Fl-ΔTAR–HalTzl_AAA_	161.2 ± 35.0

## Data Availability

The original contributions presented in this study are included in the article. Further inquiries can be directed to the corresponding author.

## Abbreviations

1,4-TzlNBA_A_	nucleobase amino acid bearing of adenine attached to the side chain of Aha via 1,4-linked 1,2,3-triazole moiety
Ac	acetyl
ACN	acetonitrile
Aha	*L*-azidohomoalanine
ARR	arginine-rich region
ASLLys3	unmodified anticodon stem–loop domain of human tRNA^Lys3^
B, BH	nucleobase
bp	base pair
C18	octadodecane chain
CuAAC	Cu(I)-catalyzed azide–alkyne cycloaddition
DCM	dichloromethane
DMAP	4-Dimethylaminopyridine
DMF	*N*,*N*-dimethylformamide
FAM(6)	fluorescein isomer 6
Fl-RNA	5′-fluorescein labeled RNA
Fmoc	fluorenylmethyloxycarbonyl
Fmoc-Aha	Fmoc-*L*-azidohomoalanine
Hal	*L*-homoalanine
HalTzl_AAA_	trinucleopeptide designed on the basis of *L*-azidohomoalanine (Hal), containing adenine attached through a 1,4-linked-triazole linker to the Hal’s side chain
HATU	[dimethylamino(triazolo[4,5-b]pyridin-3-yloxy)methylidene]-dimethylazanium;3-hydroxytriazolo[4,5-b]pyridine hexafluorophosphate
HIV-1	human immunodeficiency virus type 1
HOAt	1-Hydroxy-7-azabenzotriazole
tRNALys	tRNA specific for lysine
htRNALys3	human tRNA specific for lysine (isoacceptor type 3)
MeOH	methanol
NBA	nucleobase amino acid
NP	nucleopeptide
PBS	priming binding side
PNA	peptide nucleic acid
RNAP II	RNA polymerase II
RT	reverse transcriptase
SPPS	solid-phase peptide synthesis
ssRNA	single-stranded RNA
TAR UUU	*trans*-activation response element with UUU bulge
TB	tris/borate buffer
t-BuOH	tert-butanol
TFA	trifluoroacetic acid
Tzl	1,2,3-triazol
